# Quantum private set intersection cardinality based on bloom filter

**DOI:** 10.1038/s41598-021-96770-1

**Published:** 2021-08-30

**Authors:** Bai Liu, Ou Ruan, Runhua Shi, Mingwu Zhang

**Affiliations:** School of Computer Science, Hubei University of Technology, Wuhan, 430068 China

**Keywords:** Engineering, Mathematics and computing

## Abstract

Private Set Intersection Cardinality that enable Multi-party to privately compute the cardinality of the set intersection without disclosing their own information. It is equivalent to a secure, distributed database query and has many practical applications in privacy preserving and data sharing. In this paper, we propose a novel quantum private set intersection cardinality based on Bloom filter, which can resist the quantum attack. It is a completely novel constructive protocol for computing the intersection cardinality by using Bloom filter. The protocol uses single photons, so it only need to do some simple single-photon operations and tests. Thus it is more likely to realize through the present technologies. The validity of the protocol is verified by comparing with other protocols. The protocol implements privacy protection without increasing the computational complexity and communication complexity, which are independent with data scale. Therefore, the protocol has a good prospects in dealing with big data, privacy-protection and information-sharing, such as the patient contact for COVID-19.

## Introduction

Protecting data privacy is a very important technology, which is a legal obligation in many countries. For example, US privacy law COPPA, England Data Protection Act, Swedish Data Act, and other various of national privacy regulations. But it is still a challenging task for protecting data privacy in using and transmission. For this reason, there are many security solutions to protect privacy when data is processing or transmitting. Meanwhile, the data scale for processing and protecting is getting larger and larger. Such as, geneticists should search several billion base pairs in an individual’s genome to study genetic diseases, epidemiologists need to access to medical databases which contain records of thousands of millions patients to study risk factors for disease, Online retailers hope to increase customer satisfaction by linking their transaction records to their customers’ social networking activities. So privacy-protecting in large scale data processing brings new challenges to us: how to protect the data privacy with the large scale data processing, and how to meet the quick-speed and throughput rate of modern applications. In the era of “big data”, efficiency has become a key standard in designing privacy protection protocols.

One of the aspects of privacy protection research is about the Private Set Intersection (PSI)^1, 2^ cardinality. PSI cardinality enables multi-parties, one server and some clients, to jointly calculate the intersection cardinality with their private sets. And then the clients get the intersection cardinality and the server get nothing after processing the protocol. The main reason of PSI cardinality has been widely studied is that it has many real applications. Such as, PSI cardinality has been used in privacy preserving data mining^3^, information-sharing^4^, human genome research^5^, national security^6^, Botnet identification^7^, medical data preserving^8, 9^, social networks^10, 11^, location privacy protecting^12, 13^, searchable encryption scheme^14^ and anonymous authentication^15, 16^. In recent years more and more PSI cardinality protocols are proposed, e.g.^17–25^.etc.

In these proposed protocols, most of them are based on classical cryptography. And these protocols are often viewed as inconsistent with reality. One reason is that the efficiency and performance becomes outrageous when the input size becomes larger and larger. It’s hard to improve performance just by scaling up the hardware. The other reason is that the advent of quantum computing, the increasing power of algorithms poses a great challenge to the security of classical cryptography which is based on unconfirmed arduous hypothesis^16^.

Such criticism, however, is not without foundation. In literature^26^, the performance of the current proposed PSI cardinality protocols are compared. For example, scalable private set intersection based on OT extension by Pinkas^18^ and the private set intersection on outsourced private data sets by Aydin^27^ have high efficiency with less data, but when computing the intersection of $2^{20}$-element sets, Pinkas’s protocol needs 56738 millisecond, Aydin’s protocol needs 6864.2 seconds, and with the increasing of data scale, the efficiency decreases greatly. In addition, with the development of quantum computing, the proposed classical PSI protocols are vulnerable to attack by quantum computers. Therefore, the combination of quantum computer and cryptography has been paid more attention by scholars. For instance, quantum authentication protocol^28^, quantum protocols for secure multi-party summation^29^, quantum digital signature^30^, identity-based quantum signature^31^ and quantum private query protocols^32, 33^. Of course, there are some quantum protocols for PSI cardinality which are proposed^34, 35^ in recently. However, we need more practical and high-efficiency PSI cardinality protocols to fit the application in real world.

Contributions: In this paper, we propose a novel quantum private set intersection cardinality based on the Bloom filter, which can resist the quantum attacks. It is a completely novel constructive protocol for computing the intersection cardinality by using Bloom filter. Firstly, the elements in two data sets are filtered by using a Bloom filter, and then are transmitted by using BB84 protocol. Lastly, the intersection of privacy sets can be calculated. The novel cardinality protocol uses single photons, so it only need to do some simple single-photon operations and tests. Comparing with other protocols, the results show that the novel protocol achieves privacy preservation without increasing computational complexity and communication complexity, and the computational complexity and communication complexity are independent with the data scale. Thus it is more likely to realize with the present technologies. Therefore, the protocol has a good prospects in dealing with big data, privacy-protection and information-sharing, such as the patient contact for COVID-19.

In this paper, we present a practical and feasible quantum private set intersection cardinality protocol, which can privately compute the intersection cardinality. The organization of the paper is following, the second section is the basic knowledge about BB84 protocol and Bloom filter which will use in the protocol. We present a novel protocol about quantum private set intersection cardinality based on Bloom filter in “Quantum private set intersection cardinality” section. The security and correctness analysis are shown in “Performance” section. Finally, in “Conclusion” section, we give the conclusion of the paper.

## Preliminaries

### BB84 protocol

The BB84 protocol^36^ encodes information with four polarized photons. Let’s label these four states of polarization as $\{\leftrightarrow$, $\nearrow$, $\updownarrow$, $\nwarrow \}$. In two dimension Hilbert space $X=\{\leftrightarrow ,\updownarrow \}$ and $Z=\{\nearrow , \nwarrow \}$ form two different orthogonal basis. Based on the Uncertainty Principle, *X* can differentiate $\leftrightarrow$ and $\updownarrow$ state, *Z* can differentiate $\nearrow$ and $\nwarrow$ state.

The following four steps are the BB84 protocol.

(1) Coding and Transmission. The sender, Alice, randomly selects a basis from *X* and *Z* and encodes the information. Then Alice records the basis that she has selected.

(2) Reception and test. The receiver, Bob, randomly selects a basis from *X* and *Z* and tests its receiving state. Then Bob records the basis.

(3) Comparison and selection. Bob tells Alice the bases he have chosen, Alice responses on which bases they have selected the same. Then they discard the other different bits. By this means, they can share a key which is called row key.

(4) Testing of Eavesdropping. Alice and Bob randomly select some bits in row key and compare them in classical channel. If there exist error bits, it means the key is not secure and exists an eavesdropper.

The probability which Alice and Bob select the same basis is 1/2, so the efficiency will be $50{\%}$. If there is an eavesdropper who wants to test the states by using the random basis, He will have 1/2 possibility to select the correct basis. However, the eavesdropper selects the incorrect basis, he will alter the state. If Bob options the correct basis, he will get an incorrect bit. So each time when the eavesdropper tests, he has 1/4 possibility to get wrong bit. when Alice and Bob select n bits to test whether there exist an eavesdropper, the possibility will be $1-(3/4)n$ with the eavesdropper being detected.

### Bloom filter

Bloom filter^37^is a space and time efficient method, which can test an element whether in a set or not. An initial Bloom filter *b* includes *m* bits that the initial values are 0s, and has *k* hash functions $h_i(0\le i<k)$. Here we could get the *k* hash functions from random oracles. And $b_j (0\le j< m)$ is the *j*-th bit of the Bloom filter *b*. Bloom filter has two kinds of operations, one is Add(*x*), the other is *Test*(*x*). Add(*x*) adds element *x* to the set. *Test*(*x*) tests the element *x* to the set.

*Create*(*m*): *m* bits $(0\le j< m)$ are set to 0

1 $$\forall j\cdot b_j=0$$

and *k* hash functions $h_i(0\le i<k)$

2 $$\forall i\cdot h_i:\{0,1\}^*\rightarrow \{0,\dots ,m-1\}$$

*Add*(*x*): Hash the element *x* by using the *k* hash functions $h_i$ and change the *k* bits $g_i$ to 1.

3 $$\forall i\cdot g_i=h_i(x)\Longrightarrow b_{g_i}=1$$

*Test*(*x*): Using all *k* hash functions $h_i$ to hash the element *x* and judging all *k* bits $g_i$ in set, then the test function returns 1 (true).

4 $$\bigwedge _{i=0}^{k-1}b_{h_i}(x)$$

However, due to the collision probability of the hash function, it is impossible to guarantee that the element must exist in the set when the element$'$s $b_i$ are all 1. So it may be exist a certain false positive probability in Bloom filter, namely the false positive rate. i.e. *Test*(*x*) may be true, but *x* is not added in set. The more data adds into set, the larger false positives. The maximum false positive rate will be $2^{-k}$, when *w* elements are added into the set, then the size m of the Bloom filter could be computed

5 $$m=\frac{wk}{ln^{2}2}$$

### Quantum private set intersection cardinality

Here, we give the definition of quantum private set intersection cardinality(QPSI).

#### **Definition 1**

Quantum private set intersection cardinality(QPSI), there are two clients with the input of private set *A* and *B*. After running QPSI protocol, the client can get nothing except the intersection cardinality$|A\cap {B}|$. In addition, QPSI should satisfy the following privacy requirements:

Client *A* privacy: The client *A* learns no information about other sets except the intersection $|A\cap {B}|$.

Client *B* privacy: The client *B* learns no information about other sets except the intersection $|A\cap {B}|$.

Fairness: client *A* and client *B* are two equal entities, and they cannot through cheating with each other to get the private information. Finally, client *A* and client *B* get the result of $|A\cap {B}|$ with equal chance.

Here, we introduce a third party (Charlie) to assist client Alice and client Bob to calculate the intersection cardinality with the input private sets, and then propose a novel QPSI protocol based on Bloom filter with the help of Charlie. Charlie could be dishonest but never collude with other parties.

## Quantum private set intersection cardinality

### System model

**Figure 1:**
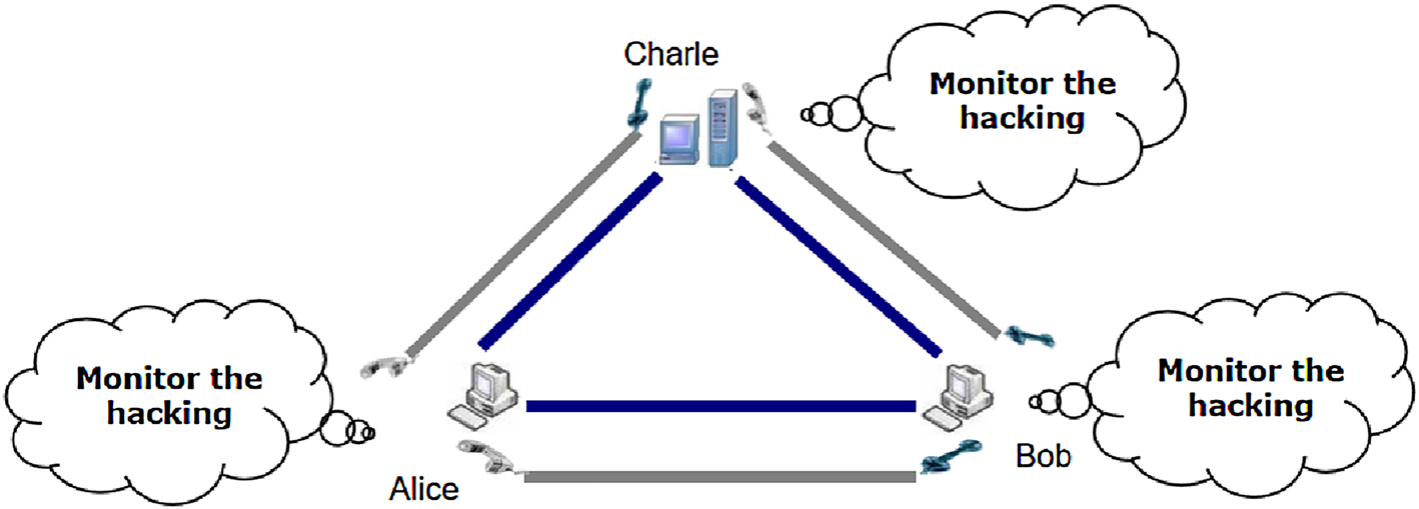
System model

Based on the quantum public key distribution, BB84 protocol and Bloom filter, we propose a novel QPSI protocol to calculate the intersection cardinality with the input private sets. First we assume that the system model has a third party (Charlie) and two clients(Alice and Bob), and the sets *A*, *B* are the private sets of Alice and Bob. The elements in *A*, *B* lie in $Z_N$, where $Z_N=\{0,1,2,\dots ,N-1\}$, $N=2^n$ (i.e.$n=log{N}$). Moreover, assume that $\sum _{i=1}^nn_{c_i}<\frac{N}{2}$, *N* and $n_{c_i}$ are public. In the protocol, we suppose all the clients and the third party are semi-honest: they are curious with the privacy of others, but are honest to carry out the operations of the scheme. The system shows in Fig. 1.

### Protocol

The protocol includes Thirteen steps as following:

Step 1. Alice initials the bloom filiter, generates the the bloom filiter(*N*) and *k* hash functions.

Step 2. By running BB84 QKD protocol, Alice shares the *k* hash functions $h_i$ and *N* with Bob.

Step 3. Alice and Bob use the *k* hash functions $h_i$ to hash the private sets *A*, *B* into the corresponding private vectors $(x_{0}, x_{1}, \dots , x_{N-1})$, $(y_{0}, y_{1}, \dots , y_{N-1})$ respectively.

Alice generates the private vector $(x_{0}, x_{1}, ...,x_{N-1})\in F^{N}_{2}$ by her private set *A*, where each element of the set determines one component of the vector. Similarly, Bob generates the private vector $(y_{0}, y_{1}, \dots , y_{N-1})\in F^{N}_{2}$ by his private set *B*.

Step 4. Charlie chooses *N* groups of single photon sequences, and each group includes *m* single photons, these single photons are chosen randomly from the following four states, $\{|{0^{\prime}}\rangle ,|{1^{\prime}}\rangle ,|{+^{\prime}}\rangle ,|{-^{\prime}}\rangle \}$,

6 $$|{0^{\prime}}\rangle = cos\theta |{0}\rangle +sin\theta |{1}\rangle$$

7 $$|{1^{\prime}}\rangle = sin\theta |{0}\rangle -cos\theta |{1}\rangle$$

8 $$|{+^{\prime}}\rangle =\frac{|{0^{\prime}}\rangle +|{1^{\prime}}\rangle }{\sqrt{2}}$$

9 $$|{-^{\prime}}\rangle =\frac{|{0^{\prime}}\rangle -|{1^{\prime}}\rangle }{\sqrt{2}}$$

Assume $\theta \in (0,\frac{\pi }{4})$, we find the best result is $\theta =\frac{\pi }{8}$ and $m=logN$. Here, *N* groups of single photon are $\{s^{1}_1,s^{1}_2,\dots ,s^{1}_m\}$, $\{s^{2}_1,s^{2}_2,\dots ,s^{2}_m$;$\dots$;$s^{N}_1,s^{N}_2,\dots ,s^{N}_m\}$ respectively. Furthermore, we use *S* to express the whole sequence of *mN* signal photons $\{s^{1}_1,s^{1}_2,\dots ,s^{1}_m;s^{2}_1,s^{2}_2,\dots ,s^{2}_m;\dots ;$ $s^{N}_1,s^{N}_2,\dots ,s^{N}_m\}$. In addition, Charlie records the initial states of *N* groups of photon sequences that he has chosen.

Step 5. Charlie again chooses $m^*(m^*\le m)$ additional photons which are in four states $\{|0\rangle ,|1\rangle ,|+\rangle ,|-\rangle \}$, and inserts each group single photon sequences randomly. We call these photons are puppet photons which can avoid attack from the participant (such as Bob) e.g.,$\{s^{i}_1,s^{*i}_1,s^{i}_2,s^{*i}_2,\dots ,s^{i}_m,s^{*i}_m,\}$, here $s^{*i}_j$ are the puppet photons. Correspondingly, we use $S^*$ to denote the sequence of all $(m+m^*)N$ photons, which includes $m^*N$ puppet photons and *mN* signal photons. Charlie makes a record of the positions where these puppet photons have inserted.

Step 6. Charlie chooses *q* decoy photons randomly from four states $\{|0\rangle ,|1\rangle ,|+\rangle ,|-\rangle \}$. when transmitting the photon sequence, these decoy photons can check if there is an eavesdropper or not. In addition, Charlie randomly puts the *q* decoy photons into the sequence $S^*$, and calls the new sequence as $S^{*}_C$. Then Charlie records the details of the positions and states of the *q* decoy photons. Thus, only Charlie knows the initial states and the positions of the *q* decoy photons. Finally, Charlie sends the new sequence $S^{*}_C$ which include signal photons, puppet photons and decoy photons to Alice in order from quantum channel.

Step 7. When Alice receives the sequence $S^{*}_C$ from Charlie, she will ask for Charlie opening the positions of *q* decoy photons in $S^{*}_C$ and the corresponding test bases. Then Alice tests the decoy photons sequence with the right bases and publishes the corresponding test consequences. Charlie contrasts the initial states of the decoy photons that he has recorded to the corresponding test consequences of Alice. Lastly, comparing the error rate with the threshold value which is decided in advance by the channel noise, if the error rate is higher, this protocol will be discarded. Otherwise, go to the next step.

Step 8. Alice deletes the decoy photons from $S^{*}_{C}$ and obtains the photons sequence $S^*$, that includes *N* groups, and each group has $(m+m^*)$ photons, the single photon sequences are $\{s^{i}_{1},s^{*i}_{1},s^{i}_{2},s^{*i}_{2},\dots ,s^{i}_{m},s^{*i}_{m}\}$ for $i=1,2,\dots ,N$. Alice does a unitary operation on the signal photon and the puppet photon, i.e., for $s^{i}_{j}(j=1,2,\dots ,m)$ and $s^{*i}_{j}(j=1,2,\dots ,m^*)$, the strategies is that:if $x^*_{i-1}=0$, Alice will do a local unitary operation *I* on the signal photon and the puppet photon $s^*{i}_{j}(s^{*i}_{j})$; If $x^*_{i-1}=1$, Alice will do a local unitary operation $\sigma _{x}$ on the signal photon and the puppet photon $s^*{i}_{j}(s^{*i}_{j})$.

10 $$I=|0\rangle \langle 0|+|1\rangle \langle 1|$$

11 $$\sigma _x=|0\rangle \langle 1|+|1\rangle \langle 0|$$

12 $$\sigma _z=|0\rangle \langle 0|-|1\rangle \langle 1|$$

Step 9. Then, Alice chooses *q* decoy photons randomly from four states$\{|0\rangle ,|1\rangle ,|+\rangle ,|-\rangle \}$ to avoid eavesdropping. Similarly, Alice puts *q* decoy photons into the sequence $S^*$ randomly, and we call the new sequence as $S^*_A$. Then Alice records the decoy photons’ detail positions and states. Finally, Alice sends the new photons sequence $S^*_A$ to Bob in orderly through the quantum channel.

Step 10. Analogously, when Bob receives the photons sequence $S^*_A$ from Alice, he asks Alice to publish the detail positions of the *q* decoy photons in $S^*_A$ and the corresponding test bases. Then Bob tests the decoy photons sequence with the right bases and publishes the corresponding test consequences. Alice contrasts the initial states of the *q* decoy photons that he has recorded to the corresponding test consequences of Bob. Compares the error rate with the threshold value which is decided in advance by the channel noise. Thus, if the error rate is higher, this protocol will be discarded. Otherwise, go to the next step.

Step 11. Bob deletes the *q* decoy photons from $S^*_A$ and gets $S^*$, that includes *N* groups, and each group has $(m+m^*)$ photons. The photon sequences are $\{s^{i}_1,s^{*i}_1,s^{i}_2,s^{*i}_2,\dots ,s^{i}_m,s^{*i}_m,\}$, for $i=1, 2,\dots ,N$. Bob does the same unitary operation as Alice on the $(m+m^*)$ photons:$s^i_{j}$ for $j=1,2,\dots ,m^*$ and $s^{*i}_{j}$ for $j=1, 2,\dots , m^*$. The unitary operation is following: if $Test(y^*_{i-1})=0$, Bob will do a local unitary operation *I* on photons $s^i_{j}(s^{*i}_{j})$; if $Test(y^*_{i-1})=1$, Bob will do an operation $\sigma _z$ on photons $s^i_{j}(s^{*i}_{j})$.

Step 12. Analogously, Bob chooses *q* decoy photons randomly from four states $\{|0\rangle ,|1\rangle ,|+\rangle ,|-\rangle \}$ to avoid eavesdropping. Then Bob puts the *q* decoy photons into the sequence $S^*$ randomly, and obtains the new photons sequence $S^*_B$. Similarly, Bob records the decoy photons’ detail positions and states.Then he sends the sequence $S^*_B$ back to Charlie through the quantum channel. In addition, Charlie and Bob together check the states of the decoy photons to detect whether there is an eavesdropper in the quantum channel. The checking procedures is the same as the Step6 or Step9. If the quantum channel is security, Charlie will delete the puppet photons and the decoy photons of the $S^*_B$, then Charlie can obtain the initial photon sequence *S*, that includes *mN* signal photons, i.e.,$\{s^1_1,s^1_2,\dots ,s^1_m\};\{s^2_1,s^2_2,\dots ,s^N_1,s^N_m\}$. Moreover, Charlie chooses *t* as a counter, and initial $t=0$ .

Step 13. For the photon sequences $\{s^i_1,s^i_2,\dots ,s^i_m\}$, $i=1,2,\dots ,N$, Charlie measures each group of photons $s^1_1,s^1_2,\dots ,s^1_m$ by using the initial bases. That is to say, if the initially photons are in $|0'\rangle$ or $|1'\rangle$, Charlie will use the basis of $\{|0'\rangle ,|1'\rangle \}$, or else use the basis of $\{|+'\rangle ,|-'\rangle \}$. When Charlie finds the test consequence of one of photon in $\{s^{i}_1,s^{i}_2,\dots ,s^{i}_m\}$ is the same with its initial state, he will stop measuring in time and go on to next group $\{s^{i+1}_1,s^{i+1}_2,\dots ,s^{i+1}_m\}$; When Charlie finds all *m* test consequences are completely different from the initial states in this group, all the *m* test consequences are orthogonal to their initial states, thus the count $t= t+1$. If all group are completly tested, Charlie announces *t* which is the intersection of A and B,i.e., $t=|A\cap B|$.

## Analysis

### Correctness

Based on the Bloom filter from step1 to step3, using the function *Add*(*A*) and *Add*(*B*) with the *k* hash functions $h_i$, $i\in k$, we can get the vector $\{x_i\}$,$i\in {N}$ and $\{y_i\}$, $i\in {N}$. So the intersection cardinality of set *A* and *B* is equal to the number of $i\in {N}$ which is satisfying both $x_i=1$ and $y_i=1$, i.e., $|A\cap B|=\sum _{i=0}^{N-1}x_i\cdot y_i$.

In addition, on account of *N* components of the private vectors $(x_0,x_1,\dots ,x_{N-1})$ and $(y_0,y_1,\dots ,y_{N-1})$, Charlie chooses *N* groups of single photons, that each group includes *m* signal photons, to totalize the number which is satisfying both $x_i=1$ and $y_i=1$. It means that these *N* groups of single photon sequences can decide if it satisfies both $x_i=1$ and $y_i=1$. Here, all *m* signal photons sequences in *N* groups are selected initially in state $|0'\rangle =cos\theta |0\rangle +sin\theta |1\rangle$. In table *I*, we give all possible cases of Charlie’s test. For instance, if $x_i=1$, Alice will do the unitary operator $\sigma _x$ on the group of the *m* signal photon. So this group signal photon of the state will be changed into the state $sin\theta |0\rangle +cos\theta |1\rangle$. Just like Alice, if $y_i=1$, Bob will do the unitary operator $\sigma _z$ on the group of the *m* signal photon, then he can get the state of each signal photon in $sin\theta |0\rangle -cos\theta |1\rangle$. Therefore, the test result of this group signal photon in the end must be $|1'\rangle$, i.e., thus we can see that the final state is orthogonal to the initial state $|0'\rangle$. Then, $t=t+1$. Moreover, there are other 3 cases (i.e., it is depicted in Table 1), for example, Charlies gets the final state $|0'\rangle$ are 1 with the probabilities $(cos\theta ^2-sin\theta ^2)^2$ and $4cos\theta ^2sin\theta ^2$.

**Table 1:** The test results

$x_i$	$y_i$	Alice	Bob	The state after doing operators	Test base	The probability of test results	
						$|0'\rangle$	$|1'\rangle$
1	1	$\sigma _x$	$\sigma _z$	$sin\theta |0\rangle -cos\theta |1\rangle$	$\{|0'\rangle ,|1'\rangle \}$	–	1
1	0	$\sigma _x$	*I*	$sin\theta |0\rangle +cos\theta |1\rangle$	$\{|0'\rangle ,|1'\rangle \}$	$4cos\theta ^2sin\theta ^2$	$(cos\theta ^2-sin\theta ^2)^2$
0	1	*I*	$\sigma _x$	$cos\theta |0\rangle -sin\theta |1\rangle$	$\{|0'\rangle ,|1'\rangle \}$	$(cos\theta ^2-sin\theta ^2)^2$	$4cos\theta ^2sin\theta ^2$
0	0	*I*	*I*	$cos\theta |0\rangle +sin\theta |1\rangle$	$\{|0'\rangle ,|1'\rangle \}$	1	–

Visibly, for the first row in table*I*, the probability that the initial state is identical with Charlie’s test result is $100\%$, so in this group Charlie do one test on any signal photon and the counter *t* need not add one. In table *I*, for the second and third rows, we can know that the best choice is $\theta = \frac{\pi }{8}$ in our protocol, so that $(cos\theta ^2-sin\theta ^2)^2=(cos2\theta )^2=\frac{1}{2}$ and $4cos\theta ^2sin\theta ^2=(sin2\theta )^2=\frac{1}{2}$. It means that the probabilities of Charlie’s getting the state $|0'\rangle$ are both $\frac{1}{2}$ in the second and the third rows. Moreover, in this group the probability is $\frac{1}{2^m}$ when all test results are $|1'\rangle$, $\frac{1}{2^m}$ is small enough, it can negligible when $m\gg 2$. For instance, if $m=10$, $\frac{1}{2^10}\approx 9\cdot 766\times {10^{-4}}$; if $m=20$, $\frac{1}{2^20}\approx 9\cdot 537\times {10^{-7}}$.

So, if $x_i=1$ and $y_i=1$, in this group all test results of *m* photons will be fully disparate from the initial states. Nevertheless, if $x_i=0$ or $y_i=0$, Charlie finds that in this group at least one test result is identical with the initial state with probability $1-\frac{1}{2^m}$. It means that the error probability(i.e., “$x_i=0$ or $y_i=0$” will be judged as “$x_i=1$ or $y_i=1$”) is $\frac{1}{2^m}$. Therefore, if it has *r* errors, the error probability will be

13 $$p(t,r,m)=C^{r}_{t}\cdot 2^{-rm}$$

Here *t* is result number which contains *r* errors. So $|A\cap B|$ should be $t-r$. With different values *r*, *t* and *m*, we get the corresponding probability of *p*(*t*, *r*, *m*), and the error probability is little, if $m\approx 10$, it is can negligible. Moreover, in Eq. (13), let $m=log{t}$, then get $p(t,r,m)=C^{r}_{t}\cdot 2^{-rm}=\frac{t(t-1)(t-2)\dots (t-r)}{r!}\cdot 2^{-rm}=\frac{t(t-1)(t-2)\dots (t-r)}{r!t^r}$; if $r=2$ and $t=20$, $p(t,r,m)=\frac{t(t-1)}{2t^2}=0\cdot 475$; if $r=3$ and $t=20$, $p(p,t,r,m)=\frac{t(t-1)(t-3)}{6t^3}=0\cdot 1425$; if $r=4$ and $t=20$, $p(t,r,m)=\frac{t(t-1)(t-3)}{6t^3}=0\cdot 0303$. Let $m=log t$, then we can get the negligible error. And $t\le N$, i.e., $m\le log N$. In fact, let $m=log N$ to overcome the loss of the photons which case by environmental interference. In summary, each group of photon sequences contains *m* signal photons to ensure the correctness of the protocol.

### Security

Now we analyze the security. The protocol is implemented with the help of Charlie(TP), who could insincere but never collude with any other^34^. Firstly, we consider the Charlie’s (TP) attacks.

In order to get the partial or whole private information of Alice or Bob, insincere Charlie may initially use some entangled photon pairs (e.g., EPR pairs) to replace the initial single photons. Then Charlie will keep one photon of the entangled photon pair in his hand and send the other to Alice or Bob. When Alice or Bob receives the entangled photon, they will do the private operations (*I*, $\sigma _x$ or $\sigma _z$) on the photons, then Charlie wants to find out the operations that Alice or Bob has performed on the corresponding photon when the photon in their hands. In fact, no matter what operation Alice or Bob have done, the reduced density matrix of the subsystem that Charlie holds doesn’t change anything. For instance, if Charlie prepare the entangled photon pairs state $\frac{1}{\sqrt{3}}|01\rangle +\frac{\sqrt{2}}{\sqrt{3}}|10\rangle$, then he will keep the first photon in his hand and send the second photon to the parties, then the parties do the operations, the reduced density matrix which Charlie still keep the state $\frac{1}{3}|0\rangle \langle 0|+\frac{2}{3}|1\rangle \langle 1|$, no matter what operations the parties do. That means, Alice or Bob’s private operations can’t affect the reduced density matrix of the subsystem. So even if Charlie prepare a entangled quantum resource to replace the single photon, he would not be able to extract any of Alice’s or Bob’s private information.

In addition, if Charlie is fraudulent, he want to intercept all photons of the sequence $S^*_A$ which are send from Alice to Bob, including signal photons, puppet photons and decoy photons, and want to get some or all information about Alice’s private operations (*I*, $\sigma _x$ or $\sigma _z$) which connect with Alice’s private vector ($x_i=0$ or $x_i=1$). To avoid detection, he might just pick a particular photon from each group and instead of it with a false photon. In addition, we suppose that Charlie can accurately speculate the photon’s initial state not the decoy photon’s, then Charlie can use the optimal Unambiguous State Discrimination(*USD*) test^38^. Based on *USD* Charlie can know the select photon which the two possible states is actually in. The successful probability of *USD* is following

14 $$p^{USD}=1-F(\rho _0,\rho _1)$$

Here $F(\rho _0,\rho _1)$ is fidelity that Charlie is trying to distinguish from the two quantum states. Assuming that the initial state that Charlie send is in $|0'\rangle =cos\theta |0\rangle +sin\theta |1\rangle$, then Alice return to the state in $|0''sin\theta \rangle +cos\theta |1\rangle$ (i.e., $x_i=1$), the successful probability of *USD* is $p^{USD}$.

15 $$\begin{aligned} p^{USD}&=1-F(\rho _0,\rho _1)\\&=1-|\langle 0''|0'\rangle |\\&=1-|2sin\theta cos\theta |\\&=1-|sin2\theta | \end{aligned}$$

When $$\theta =\frac{\pi }{8}$$, get,

16 $$p^{USD}=1-|2sin\theta cos\theta | =1-|sin2\theta | =1-\frac{\sqrt{2}}{2} \approx {0.29}$$

So according to the optimal Unambiguous State Discrimination, Charlie can successfully infer $x_i=0$ or $x_i=1$ with the probability $0\cdot 29$. Whereas, Charlie still cannot get the values of any $x_i$ without the hash functions $h_k$. Since $(x_1,x_2,\dots ,x_{N-1})$ is corresponding to *ADD*(*x*) with $h_i(A)$, Charlie cannot rightly guess *i* whether belongs to Alice’s private set *A* without the information of $h_i(A)$.

Charlie tests all the photons that Alice sends to Bob directly (In fact, Alice and Bob are easily to find this malicious attack), but he can not get the information about *A* or *B*. Suppose Charlie succeeds in getting Alice’s private vector $(x_0,x_1,\dots ,x_{N-1})$, he cannot obtain the original set *A* without the hash functions $h_k$, so the security is guaranteed by hash functions $h_k$ based on Quantum Key Distribution. Clearly, the hash function $h_k$ are completely secure. Similarly, based on the *Test*(*y*) with $h_i(B)$, Bob can get the private vector $(y_0,y_1,\dots ,y_{N-1})$, and Charlie can not get Bob’s original vector $(y_0,y_1,\dots ,y_{N-1})$ because of the privacy hash function $h_k(B)$.

In consequence the protocol is esistant to attacks by a insincere or malicious Charlie.

Then, we discuss Alice’s or Bob’s attack. Assume that Bob wants to get Alice’s private input. When Bob receives the sequence $S_A$, he will delete all decoy photons in Step 11 and get the sequences $S^*$, that including Alice’s private information, Bob would not obey the rules honestly, he will try to get Alice’s vector $x_0,x_1,\dots ,x_{N-1}$ through testing $S^*$ sequences group by group, and then he sends the fake sequences to Charlie. Here, we only analyze one group photon sequences, for example, Alice send ${s^i_1,s^{*i}_1,s^i_2,\dots ,s^{*i}_2,\dots ,s^{*i}_{m*},s^i_m}$ to Bob, when Bob receives the sequences, he will hide the value of $x_i$. Moreover, suppose that the states of photons sequences ${s^i_1,s^{*i}_1,s^i_2,\dots ,s^{*i}_2,\dots ,s^{*i}_{m*},s^i_m}$ choose from $|0'\rangle =cos\theta |0\rangle +sin\theta |1\rangle$ and the states of puppet photons sequences choose from ${|0\rangle ,|1\rangle ,|+\rangle ,|-\rangle }$ by Charlie randomly. Then if the puppet photons are not considered, we can get the following two cases:

Firstly, if $x_i=0$, after Alice does the operation, the states of all signal photons are not change. Thus Bob can accurately identify $x_i$ through testing all signal photons with base ${|0'\rangle ,|1'\rangle }$, but he doesn’t know the correct base of test. So the probability that Bob know $x_i=0$ is $\frac{1}{2}$.

secondly, if $x_i=1$, after Alice does the operation $\sigma _x$ or $\sigma _z$ on photon sequences, and then the state will be changed into $|0''\rangle =sin\theta |0\rangle +cos\theta |1\rangle$ or $|1''\rangle =cos|0\rangle -sin\theta |1\rangle$. Moreover, if Bob chooses the right base ${|0'\rangle ,|1'\rangle }$ to test the photons sequences, then he is able to find the states of the signal photons which are not in the same state, and further, he could correctly understand and deduce $x_i=1$. Similarly, he doesn’t know the right test base. So the probability that Bob know $x_i=1$ is $\frac{1}{2}$.

From above analysis, if Bob is able to distinguish puppet photons and signal photons, then Bob can get the values of $x_i$ with the probability $50\%$. But, because Bob doesn’t know the states of the puppet photons and signal photons, and also doesn’t know the location that the puppet photons are inserted in the sequence of signal photons. Meanwhile, the states of any puppet photon and signal photon are non-orthogonal. Based on the basic laws of quantum mechanics, we know that the non-orthogonal states are not distinguishable. Therefore, Bob attack is not feasible.

In addition, in order to improve the security, Charlie can dynamically choose $\theta$ one group by another, where $\theta \in (0,\frac{\pi }{4}))$ , the initial states $|0'\rangle =cos\theta |0\rangle +sin\theta |1\rangle$, $|1'\rangle =sin\theta |0\rangle -cos\theta |1\rangle$. But because Bob doesn’t know the signal photons’s initial states, he could not choose the right test base yet. Therefore, he could not get the private information that Alice has encoded on the signal photons.

Lastly, we discuss the attacks from outsider. In addition, because the outsider does not know the decoy photons’s inserted positions and the test bases, if there is an eavesdropper, it will be easily to find based on the decoy photons. For example, the entangle-and-measure, the intercept-and-resend, the measure-and-resend attack are easily found by checking the decoy photons. Here we only discuss entangle-and-measure attack. Moreover, we use the decoy photons to check the eavesdropper, here the decoy photon in state $|\psi \rangle _d$, $|\psi \rangle _d\in _R\{|0\rangle ,|1\rangle ,|+\rangle ,|-\rangle \}$. When the outsider gets the decoy photons, he will use an ancillary photon with state $|0\rangle _a$ and do an oracle operator $U_f$ on ancillary photon state $|\psi \rangle _d$ and decoy state$|0\rangle _a$, the operator $U_f$ is following^39^.

17 $$\begin{aligned} \begin{aligned} U_f:|x\rangle |y\rangle \rightarrow |x\rangle |y\oplus f(x)\rangle \end{aligned} \end{aligned}$$

Thus, gets

18 $$\begin{aligned} \begin{aligned} \langle y|\langle x|U^+_{f}U_f|x\rangle |y\rangle =\langle f(x)\oplus y|\langle x|x\rangle |y\oplus f(x)\rangle =1 \end{aligned} \end{aligned}$$

Here $U^+_{f}U_f=I$, it meets the unitarity. So based on the decoy photon state $|0\rangle$ or $|0\rangle$, it can get

19 $$\begin{aligned} \begin{aligned} U_f|\psi \rangle _d|0\rangle _a&=\\ {\left\{ \begin{array}{ll} |0\rangle _d|0\oplus f(a)\rangle _a=|0\rangle _d|f(a)\rangle _a, &{} if {|\psi \rangle _d=|0\rangle _d}\\ |1\rangle _d|0\oplus f(1)\rangle _a=|1\rangle _d|f(1)\rangle _a, &{} if {|\psi \rangle _d=|1\rangle _d} \end{array}\right. } \end{aligned} \end{aligned}$$

Then, based on the state of decoy photon $\frac{|0\rangle +|1\rangle }{\sqrt{2}}$ or $\frac{|0\rangle -|1\rangle }{\sqrt{2}}$, it will get

20 $$\begin{aligned} \begin{aligned} U_f|\psi \rangle _d|0\rangle _a&=\frac{U_f|0\rangle _d|0\rangle _a\pm U_f|1\rangle _d|0\rangle _a}{\sqrt{2}}\\&=\frac{|0\rangle _d|0\oplus f(0)\rangle _a\pm |1\rangle _d|0\oplus f(1)\rangle _a}{\sqrt{2}}\\&=\frac{|0\rangle _d|f(0)\rangle _a\pm |1\rangle _d|f(1)\rangle _a}{\sqrt{2}}\\&=\frac{1}{\sqrt{2}}[\frac{|0\rangle _d+|1\rangle _d}{\sqrt{2}}\otimes \frac{|f(0)\rangle _a\pm |f(1)\rangle _a}{\sqrt{2}}\\&\quad +\frac{|0\rangle _d-|1\rangle _d}{\sqrt{2}}\otimes \frac{|f(0)\rangle _a\mp |f(1)\rangle _a}{\sqrt{2}}] \end{aligned} \end{aligned}$$

So, based on Eq. (16), the outsider can adjudicate whether it is in $|0\rangle$ or $|1\rangle$ without being detected by testing the ancillary state from the decoy photon state in $|0\rangle$ or $|1\rangle$. Moreover, based on Eq. (17), the outsider have $50\%$ probability to detect the ancillary state from the decoy photon state in $\frac{|0\rangle +|1\rangle }{\sqrt{2}}$ or$\frac{|0\rangle -|1\rangle }{\sqrt{2}}$. We know there are *q* decoy photons, so the secure requirements is determined by the *q* secure parameter. Thus, it is not feasible for outsider to carry out such attack.

In fact, the outsider stealing Alice or Bob’s information is equivalent to find the operation what Alice or Bob has done on the sequences of puppet photons and signal photons. Since the operation is based on the private vectors of Alice and Bob. So the initial states which are randomly chosen from $\{|0'\rangle , |1'\rangle , |+'\rangle , |-'\rangle \}$ and $\{|0\rangle ,|1\rangle ,|+\rangle ,|-\rangle \}$ are not knew for outsider. According to the law of quantum mechanics, we can’t distinguish the non-orthogonal states. So, the attack from outsider is not possible.

## Performance

In our QPSI protocol, we use single photons, single photons operations $I,\sigma _x,\sigma _z$ and photons tests. Single photons includes signal photons and decoy photons, signal photons’s state in $|0'\rangle ,|1'\rangle ,|+'\rangle ,|-'\rangle$, decoy photons’s state in $|0\rangle ,|1\rangle ,|+\rangle ,|-\rangle$. So the protocol is more suitable to implement than entangled states, other complex tests and operations.

Based on the BB84 protocol and literature^40^, we know that both the communication and computation complexities are *O*(*NlogN*) (here one group photons’s number is $m=logN$). Thus we know that they are independent with the data scale of set A and B. So the protocol is more suitable to handle big data.

Table 2 provides a comparison and summary of the performance with other existing protocols. In Table 2, *s* in classic algorithms represent the data scale. Table 2 shows: (1) Comparing with the classic PSI-CA protocols, The computational complexity and communication complexity will increase linearly with the increasing data scale, such as Huang’s^11^ scheme, Dong’s^25^, Kerschbaum’s^39^, and Zhu’s^41^. So if the data scale are too large, the complexity will increase linearly and the efficiency will be greatly reduced. But in our protocol the computational complexity and the communication complexities are independent with data scale. (2) Comparing with the quantum protocol of PSI-CA protocol, our protocol only uses the single photons, and adopts the single-photon operations, and tests which are more feasible with current technologies than entangled states. Such as the protocol^34^ which use the multi-photon entangled states, complicated oracle operations and tests in high dimensional Hilbert space. (3) Comparing with the exist protocols, Our protocol doesn’t have failure rate. From the above analysis, our protocol is more feasible and practical with existing technologies.

**Table 2:** Comparison of protocols in complexity

	Dong et al.	Huang et al.	Kerschbaum et al.	Zhu et al.	Shi et al.	Our protocol
Computational complexity	*O*(*s*)	*O*(*slogs*)	*O*(*s*)	*O*(*s*)	*O*(*mlogN*)	*O*(*NlogN*)
Communication complexity	*O*(*s*)	*O*(*slogs*)	*O*(*s*)	*O*(*s*)	*O*(*mlogN*)	*O*(*NlogN*)

## Conclusion

In this paper, we propose a novel quantum private set intersection cardinality based on Bloom Filter to privately compute the cardinality intersection. In order to keep the fairness, the protocol need the help of the third party (Charlie). We use basic laws of quantum mechanics to guarantee the security. Such as, the BB84 protocol and the quantum tests technology can resist all kinds of quantum attacks(the entangle-and- measure, the intercept-and-resend, the measure-and-resend attack and so on). In addition, the new protocol takes single photons as quantum resources, so we only do the simple single-photon operations and tests. Thus it is more feasible to prepare these quantum resources and do the single-photon operations and tests with present technologies. Comparing with other protocols, the results show that our protocol achieves privacy preservation without increasing computational complexity and communication complexity, and the computational complexity and communication complexity are independent with the data scale. Therefore, our protocol has a good prospect in dealing with big data, privacy-protection and information-sharing.
